# Artificial Intelligence for Hospital Health Care: Application Cases and Answers to Challenges in European Hospitals

**DOI:** 10.3390/healthcare9080961

**Published:** 2021-07-29

**Authors:** Matthias Klumpp, Marcus Hintze, Milla Immonen, Francisco Ródenas-Rigla, Francesco Pilati, Fernando Aparicio-Martínez, Dilay Çelebi, Thomas Liebig, Mats Jirstrand, Oliver Urbann, Marja Hedman, Jukka A. Lipponen, Silvio Bicciato, Anda-Petronela Radan, Bernardo Valdivieso, Wolfgang Thronicke, Dimitrios Gunopulos, Ricard Delgado-Gonzalo

**Affiliations:** 1 Fraunhofer Institute for Material Flow and Logistics (IML), Josef-von-Fraunhofer-Str. 2-4, 44227 Dortmund, Germany; marcus.hintze@iml.fraunhofer.de (M.H.); oliver.urbann@iml.fraunhofer.de (O.U.); 2 Department of Business Administration, Georg-August-University of Göttingen, Platz der Göttinger Sieben 3, 37073 Göttingen, Germany; 3 VTT Technical Research Centre of Finland Ltd., Kaitoväylä 1, 90571 Oulu, Finland; milla.immonen@vtt.fi; 4 Polibienestar Research Institute, University of Valencia, Carrer del Serpis 29, 46022 València, Spain; francisco.rodenas@uv.es; 5 Department of Industrial Engineering, University of Trento, Via Sommarive 9, 38123 Trento, Italy; francesco.pilati@unitn.it; 6 NUNSYS S.L., Calle Gustave Eiffel 3, 46980 Valencia, Spain; fernando.aparicio@nunsys.com; 7 Department of Management Engineering, Istanbul Technical University, Macka, Beşiktaş, 34367 İstanbul, Turkey; celebid@itu.edu.tr; 8 TU Dortmund, Artificial Intelligence Unit, Otto-Hahn-Straße 12, 44221 Dortmund, Germany; thomas.liebig@tu-dortmund.de; 9 Materna Information & Communications SE, Artificial Intelligence Unit, Voßkuhle 37, 44141 Dortmund, Germany; 10 Fraunhofer-Chalmers Centre & Fraunhofer Center for Machine Learning, Chalmers Science Park, 41288 Gothenburg, Sweden; matsj@fcc.chalmers.se; 11 Heart Center, Kuopio University Hospital and Institute of Clinical Medicine, University of Eastern Finland, Ritva Jauhiainen-Bruun, 70029 Kuopio, Finland; marja.hedman@kuh.fi; 12 Department of Applied Physics, University of Eastern Finland, Yliopistonranta 1, 70210 Kuopio, Finland; jukka.lipponen@uef.fi; 13 Interdepartmental Center for Stem Cells and Regenerative Medicine (CIDSTEM), Department of Life Sciences, University of Modena and Reggio Emilia, Via Gottardi 100, 41125 Modena, Italy; silvio.bicciato@unimore.it; 14 Department of Obstetrics and Gynecology, University Hospital of Bern, Murtenstraße 11, 3008 Bern, Switzerland; Anda-Petronela.Radan@insel.ch; 15 La Fe University Hospital Valencia, Avinguda de Fernando Abril Martorell 106, 46026 València, Spain; valdivieso_ber@gva.es; 16 ATOS Information Technology GmbH, Fürstenallee 11, 33102 Paderborn, Germany; wolfgang.thronicke@atos.net; 17 Department of Informatics and Telecommunications, National and Kapodistrian University of Athens, Panepistimioupolis, Ilisia, 15784 Athens, Greece; dgunopulos@gmail.com; 18 Centre Suisse d’Électronique et de Microtechnique CSEM, Jaquet Droz 1, 2002 Neuchâtel, Switzerland; ricard.delgado@csem.ch

**Keywords:** COVID-19, artificial intelligence, uses cases, European hospitals, benefits

## Abstract

The development and implementation of artificial intelligence (AI) applications in health care contexts is a concurrent research and management question. Especially for hospitals, the expectations regarding improved efficiency and effectiveness by the introduction of novel AI applications are huge. However, experiences with real-life AI use cases are still scarce. As a first step towards structuring and comparing such experiences, this paper is presenting a comparative approach from nine European hospitals and eleven different use cases with possible application areas and benefits of hospital AI technologies. This is structured as a current review and opinion article from a diverse range of researchers and health care professionals. This contributes to important improvement options also for pandemic crises challenges, e.g., the current COVID-19 situation. The expected advantages as well as challenges regarding data protection, privacy, or human acceptance are reported. Altogether, the diversity of application cases is a core characteristic of AI applications in hospitals, and this requires a specific approach for successful implementation in the health care sector. This can include specialized solutions for hospitals regarding human–computer interaction, data management, and communication in AI implementation projects.

## 1. Introduction

Research into applications of artificial intelligence (AI) in health care and within hospitals is a crucial area of innovation [1]. Smart health care with the support of AI technologies, such as Machine Learning (ML), is needed due to specific challenges in the provision of medical support in European countries as well as in the rest of the world. It is not only the outbreak of the COVID-19 pandemic that reveals the current problems and challenges facing European hospitals. The success in the science of medicine in the last decades has had the effect of patients becoming older, frailer, and multi-morbid due to a longer lifetime expectation [2].

This is accompanied by the fact that medical care and diseases are becoming increasingly complex. Due to this medical complexity, medical personnel are becoming more and more specialized, which cannot in general be fully provided for by smaller hospitals in rural areas. Added to this is the demographic change already emerging in Europe, e.g., the population of over 80-year-olds in the EU27 will double from 6.1% in 2020 to 12.5% in 2060 [3]. Hence, more older people with their specific health problems will use the health care system. In contrast to this, the number of young well-trained medical personnel is currently decreasing and a shortage of skilled personnel, such as doctors and nurses, is already emerging in many European nations [4].

The challenges of the simultaneous increase of older and multi-morbid patients with complex diseases and the shortage of skilled personnel are also hampered by the increasing economic constraints on hospitals. An increase in chronic diseases due to aging populations and shortage of medical specialists results in resource scarcity and medical sustainability challenges. In order not to endanger the living and health standards of the European nations it will be necessary to develop applied AI-solutions to relieve the burden of increased workload as well as being instrumental to deliver efficient, effective, and high-quality health care.

Adaptability and agility at hospitals are major prerequisites in this context, and narrowing the application of AI to optimization solely does miss the point in many cases. By opening a wider range of actionable options, from personalized medical diagnosis and treatment to choices in care, sourcing, and logistics areas, AI applications will provide more important support avenues than efficiency enhancements only [5,6]. In addition, multiple benefits regarding the ongoing COVID-19 pandemic can also be expected and should be further explored, especially regarding data analysis and preventing unnecessary patient contact for health care personnel in hospitals as centres of the fight against the viral disease [7].

**Figure 1:**
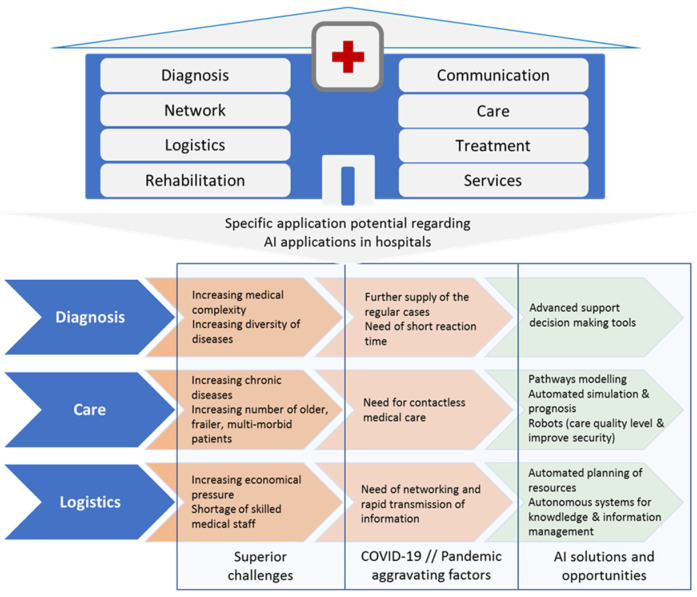
Interrelation structure of AI application areas for AI in hospitals.

AI can also contribute to the fight against pandemics as COVID-19, helping hospitals focus resources on pandemic patient’s treatments in the current as well as possible future situations. In this sense, most AI applications are directed at contactless analysis, diagnosis, and treatment (e.g., self-treatment and prevention), reducing the number of personal contacts and hospital visits, therefore reducing the potential spread of COVID-19 and other viral pandemics. AI in particular offers great potential for improving medical care and supporting the medical staff. The state of the art and the challenges regarding AI applications in hospitals and the health care sector are described for specific application areas in Figure 1.

With regards to the introduction of AI applications in hospitals, two specific questions arise, with the answers to them as the central contributions of this paper: First, what are the requirements and hospital setups for AI applications? To this end, the authors carried out a survey of different European hospitals and identified relevant projects in this field. As a result, the main fields of application of AI for hospitals are found as care, diagnosis, and logistics. The hospitals surveyed saw the greatest medical and economical potential in these three areas through the use of AI. Building on this, the paper outlines altogether 11 use cases in 9 hospitals across Europe, informing how AI can contribute to agility and efficiency in hospitals, improving health care from the resource efficiency as well as the service quality and choice side, aligned with the core hospital workflow and value adding processes. The second question is: How can a basic structure for the different AI use cases be established to avoid the mistake of developing isolated solutions that are difficult to transfer across hospitals? The authors propose three basics support areas which help to ensure a holistic approach to AI application implementation and transfer within the paper.

The paper is structured as follows: The following section is outlining the applied use case methodology for the analysis presented. The next section is describing the specific use case descriptions and expectations of hospitals towards AI applications. The following section presents a discussion regarding possible benefits and challenges as well as concept items such as human–computer interaction and medical data space concepts to overcome the challenges posed by AI applications in the hospital context. The final section provides an outlook towards future developments and challenges for AI applications in hospitals.

## 2. Use Case Methodology

The first step to identify the current challenges and areas of interest of European hospitals was to create a survey. The survey was carried out to obtain a differentiated view of the needs of European hospitals. Specifics were requested, such as country, type, number of patients and beds, and the main health care areas. In addition, hospital decision-makers identified specific areas of application and presented the focus and expected output of the utility of AI. The following Table 1 outlines the specific setup of these hospital characteristics for the institutions included in the survey.

The framework situations for the outlined AI use cases are characterized by their specific hospital setup in a broad multitude of European hospitals. By means of surveys carried out in the hospitals participating in this analysis, different health care personnel have provided systematic answers to a structured questionnaire dealing with relevant aspects to the study. The hospitals where asked to detail current practical problems in different areas, how are they currently managing these problems, ways and mechanisms to improve in these areas by means of AI, and relevant KPIs determining qualitative and quantitative improvements related to the adoption of the AI application. As a result, after extracting the information from these surveys, use cases could be drafted for the different health institutions, based on real and actual needs and opportunities. Societies require an effective and efficient health care system and especially hospitals as nodes in a network of actors providing high-quality services, resources and serving patients. The following table summarizes the main expectations as stated by the health organizations in the survey (see Table 2).

From the expectations, a total of 11 use cases in different health areas has been envisioned. It turns out that three particular fields are of specific interest to the hospitals surveyed: diagnosis, care and logistics.

In the field of diagnosis, clinical decisions still mostly depend on the application of clinical practice guidelines, instead of being based on the use of automatic decision support tools that exploit the increasing availability of medical data from molecular assays, electronic health records, clinical and pathological images, and wearable connected sensors. Nowadays, clinicians face enormous challenges in reconciling heterogeneous clinical data and exploiting the information content to make optimal decisions when assessing a disease or its progression, and this situation has become more evident in the midst of the global COVID-19 pandemic. Thus, there is an urgent need to develop smart decision support systems, which assist clinicians in making rapid and precise diagnostic decisions through the combination of multiple data sources. AI-based methodologies for medical diagnosis and medical decision support have gained attention in the recent years as these systems hold promise to automate the diagnosis and triage processes, thus optimizing and accelerating the referral process especially in urgent and critical cases. Recently, state-of-the-art examples demonstrated that software based on AI can be used in clinical practice to improve decision-making and to achieve fast and accurate databased diagnosis of various pathologies. In particular, AI has been proven particularly helpful in areas where the diagnostic information is already digitized, such as: for detection of cancers based on molecular, genomic, and radiological data [8], making individual prognosis in psychiatry using neuroimaging [9,10] identifying strokes from computed tomography scans [11], assessing the risk of sudden cardiac death or other heart diseases based on electrocardiograms and cardiac magnetic resonance images [12,13], classifying skin lesions from skin images [14], finding indicators of diabetic retinopathy in eye images [15], and detect phenotypes that correlate with rare genetic diseases from patient facial photos [16]. The change in clinical practice through and by the means of technological innovation is today decisively enabling health care systems to face to the continuous economic, socio-demographic and epidemiological pressures [17]. However, technological innovation, although important and central, must be carefully examined and accompanied to ensure that it really corresponds to effective social innovation. As addressed by MedTech Europe, developing AI systems and algorithms for healthcare settings requires specific skillsets which are in short supply, and investment in education and training of professionals involved (e.g., data scientists, practitioners, software engineers, clinical engineers), is mandatory [18].

In the field of care, AI for health has shown great potential to improve healthcare efficiency, considering the relationship between health factors, including service and management, and ICT factors that include sensors, networks, data resources, platforms, applications and solutions [19]. For the hospital facilities, AI is one of the most powerful technologies from the perspectives of data, computing power and algorithms. Research in Health 4.0 has been conducted in an interdisciplinary way with a diversified set of applications and functionalities and in terms of its implementation, it has been more commonly found in hospitals’ information flows, especially the ones related to healthcare treatments [20]. In this context, it is also necessary to consider and to assess the prevailing opinions and expectations among stakeholders regarding ICT health solutions, such as the improvement of factors that affect quality of life, quality of health care, patient’s knowledge, monetary aspects, or data security and privacy [21]. Although the research trend in the field of chronic care is to keep a continuous monitoring of each patient (promoting continuity of health and social care), tools to identify chronic patients and analyze the use of health services (care pathways) that they perform do not exist yet, and in addition there are no AI models that facilitate the design of integrated care pathways. There is clear evidence of the relevance of organization and management of the technological issue in the health care, concept further reinforced on the light of recent COVID-19 pandemic. Assessment, supply, prioritization, appropriate usage, and exploitation are indeed not a trivial duty, and the final success of any health process is widely affected by technology management issues.

In the field of logistics, AI can be applied in the forms of optimizing ML algorithms for scheduling and transportation planning [22,23,24]. This has not been extended to AI-led prognosis applications at least with empirical testing. The currently existing industry standard draws on manual processes to plan and optimize resource use. Software applications are being widely used in hospitals for this problem area, such as ORBIS, Medico or M-KIS that rely on an old architecture and non-intelligent, manual interaction with users. Even specialized software modules such as myMedis support the whole process of OR management and related resource planning but still do not use AI-based technology and thus are not able to cope with rising complexity in resource planning optimization [25,26,27]. It has been reported that AI adoption by key stakeholders such as doctors remains low [28], and that existing applications do not cater enough to the specific needs of human stakeholders that are supposed to interact with the systems [29]. Accordingly, a focus on human–computer interaction (HCI) spanning pre-design, design and post-design phases as well as catering to user, system, task, and interaction characteristics [30] holds the potential to increase AI adoption and user satisfaction [31]. While expertise in HCI has been developed in the fields of computer science [32,33], it has not been systematically applied to the hospital context.

## 3. Use Cases Descriptions and Expectations

In the field of diagnosis, we propose to advance the methods that intelligently utilize heterogeneous data from various sources and novel AI-based methods for supporting medical diagnosis and decision making inside clinics. More specifically, we propose to increase the utilization of AI-based methods in four selected use cases: diagnosing coronary artery disease (CAD), assessing fetal state during labor, diagnosing epidermolysis bullosa (a rare genetic disease) and diagnosing arrhythmias automatically. All the use cases provide heterogeneous data, which at the same time is a challenge for the medical experts to handle and on the other hand provide a possibility for the rise of novel AI-based methods in supporting diagnosis and clinical decision-making. AI-based methods also enable detection of factors in medical diagnosis that are unnoticeable for humans. Collaboration between technical and medical experts is crucial to co-create such tools to be used in clinics that are highly acceptable, highly deployed, and provide real value for patients, doctors and societies.

### 3.1. Use Case 1: Coronary Artery Disease Diagnosis

Among all routinely available diagnostic tests, coronary CT angiography (CCTA) has the highest sensitivity (95–99%) for detection of coronary artery disease (CAD), with a specificity of 64–83%, and it has recently set up as the first-hand diagnostic tool for stabile chest pain. However, after CCTA there are still several patients for whom the diagnosis and reason for symptoms remains unclear and further imaging studies (myocardial perfusion and/or invasive coronary angiography) are needed to decide the best way of the treatment. Training a ML algorithm to recognize those cases for whom further imaging is likely to provide essential information among the unclear cases with suspected CAD would improve the cost-efficiency and logistic of the diagnosis of chest pain patients. In other words, the aim would be to develop a tool for evaluating the risk of the patient to have prognostic CAD for customized clinical decision-making. The number of the patients with suspected CAD transmitted to hospital for diagnostic imaging is likely to grow in the future worldwide due to recently published clinical guidelines emphasizing the use of CCTA. For the study, a number of contemporary CCTA studies imaged and essential clinical data (age, sex, cardiovascular risk factors and medication) could be used to train a machine-learning algorithm such as Disease State Index (DSI), which is a method to quantify the probability to belonging to a certain disease population, originally developed to support clinicians in diagnosing Alzheimer’s Disease [34].

### 3.2. Use Case 2: AI Based Automatic Arrhythmia Analysis

Atrial fibrillation (AF) is the most common sustained arrhythmia and is associated with significant morbidity and adverse outcomes (stroke, heart failure, death). Overall, AF is associated with five-fold greater risk of stroke. Anticoagulation therapy has been demonstrated to reduce AF-related stroke risk significantly. Paroxysmal AF (PAF) is a self-terminating recurrent form of AF. The diagnosis of PAF is often tricky since PAF episodes can be short in duration, asymptomatic and the episode incidence can be low. It is estimated that the stroke causes total costs of EUR 45 billion/year across Europe. In European countries, 1.5 million peoples are diagnosed with stroke every year, 9 million are living with stroke and it is responsible for 9% (0.4 million) of all deaths in EU [2]. Cryptogenic stroke (CS) and transient Ischemic Attack (TIA) patients and cardiac surgery patients are the three most clinically significant patient groups where PAF is often underdiagnosed. In this use case, state of the art AI-based arrhythmia analysis algorithms are developed for PAF-screening in patients with TIA or cryptogenic stroke and detection of post-operative atrial fibrillation in cardiac surgery patients. AI-based automatic arrhythmia analysis implemented in wearable sensors enables longer monitoring time with improved patient usability and still requires minimal effort from healthcare professionals. Developing novel, AI-based non-invasive methods for PAF screening, using simple wearable ECG or PPG measurement would lead to increasing rate of PAF diagnosis in cardiac surgery, CS and TIA patients. These monitoring methods will be easily exploitable and inexpensive. The timely diagnosis of PAF has an important impact since anticoagulation may save the patient’s life or prevent stroke-related disabilities such as paralysis, aphasia and chronic pain. There is a high-cost saving potential, since one prevented stroke can save EUR 20,000 of direct medical costs and more than EUR 100,000 of indirect costs (disability-adjusted life years lost).

### 3.3. Use Case 3: Fetal State Assessment during Labour

Cardiotocography (CTG), also known as electronic fetal monitoring (EFM), is used for fetal assessment before and during labour and largely replaced the use of intermittent heart rate auscultation. Visual interpretation of CTG traces is characterized today by a great inter- and intra-observer variability with low specificity. EFM has been shown to lead to unnecessary medical interventions such as caesarean section and vaginal-operative deliveries, with the associated health consequences and economic costs. The low specificity for identifying fetal hypoxia can be partially interpreted in the context of observer variability. CTG recording is widely performed for fetal assessment during delivery and has become routine in most hospitals worldwide. A software program connected to the electrodes of the electronic fetal monitoring system (EFM) registers fetal and maternal data such as fetal heart rate and its variations, maternal heart rate, uterine contractions and fetal movements. Currently, the most specific available CTG interpretation system is the FIGO (Fédération Internationale de Gynécologie et d’Obstétrique) classification, which is most commonly used worldwide [35]. Fetal outcomes after delivery are being measured by assessing following two parameters: (1) arterial pH directly after birth (blood from the umbilical cord); (2) APGAR score assessment at 1, 5 and 10 min after delivery. This not only offers information about the fetal state, but also gives observer (obstetricians and midwives) direct feedback about previous CTG interpretation during delivery as well as prediction of fetal hypoxia/acidosis. An arterial pH under 7.15 is considered to be pathologic and is a direct indicator of fetal hypoxia. An APGAR score under 7, measured 5 min after delivery is also considered to be pathologic. APGAR as scoring system based on five fetal features—appearance, pulse, grimace, activity and respiration—providing information about the status of the new-born after delivery [36]. Considering the problematic of observer variability, four scenarios are possible when CTG interpretation is performed by obstetricians or midwives: (1) normal CTG, normal outcomes (pH/APGAR); (2) pathological CTG, normal outcomes (pH/APGAR); (3) normal CTG, pathological outcomes (pH/APGAR); (4) pathological CTG, pathological outcomes (pH/APGAR). By introducing AI interpretation, the purpose is to improve scenario 2 and 3, which will in most cases lead to avoidance of surgical interventions, since the main problem of CTG is specificity; or to performing interventions at moments where one would otherwise refrain from doing so (version 3). The AI system could provide feedback when fetal asphyxia is expected (pH < 7.15 or APGAR at 5 min < 7), as well as warnings, if applicable. The proposed AI (or ensemble of several AI instances) would help in removing the existing great inter- and intra-observer variability and would lead to a direct and positive impact on effectiveness and efficiency through: (1) decrease of unnecessary caesarean section and instrumental delivery; (2) increase of specificity for identifying fetal hypoxia; (3) decrease of unnecessary health costs derived from unnecessary surgical procedures.

### 3.4. Use Case 4: Diagnosis in Epidermolysis Bullosa, a Rare Genetic Disease

In Europe, a disease is considered rare when it affects less than 1 in 2000 people. There are more than 7000 rare diseases (RDs) worldwide, about 80% of them has a genetic origin and approximately 75% affect children. RDs are estimated to affect 350 million people globally [37]. In better-resourced countries, correct diagnosis of rare genetic diseases takes on average between 5.5 and 7.5 years. In Europe and United States, nearly half of the first diagnoses are only partially correct. The deployment of effective diagnostic procedures is hampered by the underestimation of the true disease frequency (owing to the lack of RDs’ awareness) and by an insufficient knowledge of the disease pathophysiology and natural history combined with the paucity of validated disease-specific biomarkers. Epidermolysis bullosa (EB) is a group of inherited, genetic diseases in which the skin (and the mucous membranes) is very fragile and forms severe, chronic blisters and lesions after even minor frictions or trauma. This rare genetic disorder affects all genders, ethnic and racial groups and determines either an early death or a long-term debilitating and life-threatening condition, since the severe blistering and associated scarring and deformities result in poor quality of life and reduce life expectancy. In the world there are about 500,000 persons affected by this disease and 36,000 in the European Union (EU). EB can be classified into four major subtypes, such as dystrophic EB (DEB), junctional EB (JEB), EB simplex (EBS), and Kindler Syndrome depending on the gene mutations and the level of skin cleavage [38]. Within the subtypes, EB has different severity levels and clinical manifestations. There is an urgent need to develop efficient methods for the early diagnosis of the EB subtype, the prediction of the disease progression and, consequently, the selection of individualized, precision therapeutic strategies. In this endeavour, “omics technologies”, as genomic analysis by means of next generation sequencing (NGS), have recently found applications in the diagnosis, molecular subtyping, and follow-up prediction of EB. Information retrieved from these technologies represents a substantial increase in the amount of data that can be used to support EB patients, provided that advanced computational methods are available for their integrative and combinatorial analysis. In this use case, state-of-the-art AI algorithms are developed and applied for supporting early diagnosis, sub-classification, and therapeutic stratification of EB, as an example of rare genetic disease. In particular, AI-based methods will be applied to the integrative analysis of biological (genomics, molecular, immunological, and images) and epidemiological (medical records) data with the aim to: (1) support disease and disease subtype diagnosis; (2) identify distinctive features (genomic lesions, proteins, and immunological states) associated to disease severity (biomarkers) for the prediction of disease progression; (3) detect molecular signatures for guiding patient stratification for novel means of treatment (precision therapeutics). ML algorithms can be trained to integrate phenotypic and clinical data for the prioritization of disease-related genes and mutations, for the prediction of the pathogenicity and disease clinical relevance of genetic variants, and for the identification of pathogenic variant combinations. Furthermore, AI-based methods could be used for disease comprehension and therapeutic target selection by unravelling the affected genetic and molecular players and pathways. AI and ML can be applied to detect anomalies in gene expression and to correlate transcriptional patterns with molecular mechanisms and clinical phenotypes, to learn low frequency patterns, and to deliver automated class attribution [37]. Results from these analyzes would facilitate the recommendation of optimal treatment approaches and the identification of reliable biomarkers of normal versus pathogenic states and of response to therapeutics interventions. AI methods focusing on removing the existing limitations in the correct diagnosis of EB subtypes and in the prediction of the clinical course of EB patients might achieve at least the same average accuracy as medical doctors following the latest consensus reclassification of inherited EB. The AI-based integrative analysis of biological and medical data will have a direct and positive impact on effectiveness and efficiency through: (1) decrease in the time needed for the diagnosis of the correct EB subtype and the stratification of the patient for the most effective therapeutic treatment; (2) increase in the number and efficacy of diagnostic and prognostic biomarker; (3) increase in the efficacy of selection criteria to identify patients who will benefit from ex vivo gene therapy; (4) decrease of unnecessary life-threatening conditions and health costs derived from delayed diagnosis and treatment administration.

In the field of care, AI will be applied in four other use cases: to improve the management and decision support process, specifically in the chronic care pathway and resources characterization, simulation of demand and prognosis, adverse events identification and prevention, chronic resources management support tool and monitoring of the recovery process. Novel innovative tools for simulation and prognosis would become available, projecting the demand in terms of health resources for a given characteristic population in a territory, considering temporary projections of frailty condition of population and patients. As for recovery monitoring, contactless determination of vital signs will suppose an advanced functional aspect by monitoring of all patients and not only critical cases. Patients will benefit from reduced restrictions due to cables and devices. In addition, there is a time saving for nursing staff, as they do not have to put the devices on the patient and disinfect them. Regarding prevention of adverse critical conditions, the proposed approach relies on the analysis of the entire temporal series of vital signs by means of deep neural networks and hybrid approaches.

### 3.5. Use Case 5: AI Chronic Management and Decision Support Engine

According to the data of the World Health Organization (WHO), respiratory diseases together with cardiovascular diseases are leading causes of death and disability in the world. Considering this premise, the use of case will focus on the analysis of data from chronic patients diagnosed with one of these four common pathologies: COPD, asthma, coronary heart disease (e.g., heart attack) and cerebrovascular disease (e.g., stroke). The objective would be to apply AI in the clinical context of chronic care to characterize the pathways and resources used, as well as anticipate the demand of resources in order to optimize the economic costs. ML could be then used to analyze data of patients related to clinical parameters (e.g., laboratory tests), use of resources (e.g., hospitalizations), sociodemographic data (e.g., age, gender), and quality of life, among others. The AI engine would be able to support two analysis processes: the chronic care pathway and resources characterization (stratify patients by degree of frailty and map pathways), and resources demand simulation and prognosis (according to each pathway/patient strata).

### 3.6. Use Case 6: Chronic Resources Management Support Tool

As stated by the surveyed hospitals, efficient and effective scheduling of the resources is a challenge for most hospitals. Possible resources to be scheduled are patients’ beds, material, medicament and assistance kit, medical equipment (e.g., diagnostic machines) or operating theatres. The goal would be to automatically schedule the usage of the considered resources as well as to measure and improve quantitative KPIs considered relevant for the most significant hospital metrics, e.g., cost, service level, delivery time, resource utilization, etc. To achieve this objective it is necessary to carry out the following activities: (1) translating hospital needs, often presented in a medical language, in technical concepts; (2) define the scheduling problem to be tackled by the intelligent algorithm and input data; (3) development an intelligent algorithm to automatically schedule the usage of resources and to measure quantitative KPIs over time; (4) test and validation of the intelligent algorithm using real datasets with the aim to fine-tune the procedures and selection rules implemented in the algorithm; (5) continuous learning of the intelligent algorithm by its utilization, performances and evolution of the surrounding environment.

### 3.7. Use Case 7: Adverse Events Identification and Prevention

Clinicians require support in the identification and prevention of adverse clinical conditions (ACC), as well as in identifying the main related care pathways. The technology could support the clinician in the automatic identification of ACC, such as a reaction to a new drug assumed by the patient after a change of her/his treatment plan. The AI tools could analyze data caught by vital signs monitoring systems, such as heart rate, pressure, body temperature and other data coming from the patient, such as information inferred by dialog systems based on natural language processing that would periodically interact with the patient to identify specific symptoms. Additionally, the tools would be able to support clinical staff in case a change within the care pathway is needed due. The objective would be to identify and forecast ACC for patients with non-communicable chronic diseases, particularly referring to cardiovascular diseases, by using AI. Models and tools for the automatic identification of ACC would be preliminarily realized adopting retrospective data and classic ML algorithms using current guidelines on the management of diseases of interest. Such models and tools, however, could be continuously improved, following a continuous learning approach. Successively, the prevention of ACC could be attempted by advanced classification systems, based on a combination of deep learning and reinforcement learning approaches that will analyze time series data concerning the patient condition evolution at different stages of the care pathway.

### 3.8. Use Case 8: Monitoring of the Recovery Process

Monitoring of the recovery process is a key hospital process. In order to achieve a high, continuous quality, vital parameters have to be monitored constantly. Vital parameters such as the heart rate or the respiration rate are key indicators for the current health status, urgent emergencies and the recovery process. Especially, persons with chronic diseases benefit from a continuous monitoring. In areas such as operation theatres or ICUs, there is a high coverage, whereas in normal wards or floors there is little to no coverage. The objective would be to remote determination of vital parameters such as heart rate and respiration rate for an improved recovery monitoring in a patient friendly method especially for chronic diseases. This could be realized by optical sensors with remote working mode and AI algorithms such as CNNs, BNNs or adaptive optical flow. To achieve the objective it is necessary to carry out the following activities: (1) identifying of optimal positioning of optical sensors within the hospital; (2) analysis of algorithms of remote vital parameter determination in clinical environments; (3) transfer and implementation of algorithms to the clinical setting; (4) evaluation of algorithms in clinical setting by means of reference systems, which would stayed synchronized; (5) interface protocol for transmission of vital parameters to central processing unit in the hospital. It should be guaranteed that only this meta data are transferred but not the raw data, thus protecting the privacy of the patients.

In the field of logistics, AI can be implemented for example in three different use cases as described below. The main focus is the optimization of resource use. It is expected that AI will help to better predict material consumption and needs in the whole process. Besides material consumption, transport planning is a further focus point in the field of logistics.

### 3.9. Use Case 9: Material Consumption Recognition and Prognosis

Currently, in the University Hospital in Essen as well as many other hospitals in Europe the documentation of used materials with hospital patients is a non-digital paper-pencil process consuming a lot of human work time. Therefore, digital improvements regarding automated capture system for material consumption are a prominent request in hospitals and addressed in this use case. Together with an industry partner an innovative care trolley is developed with a camera system and the complementary AI-based software using ML to recognize the consumed objects with patient processes automatically. User interaction can be implemented according to current state-of-the-art concepts. It will provide a data recognition and prognosis tool relating actual material consumption to patient cases and therefore enabling a bottom-up planning and prognosis for optimized procurement and logistics in hospitals.

### 3.10. Use Case 10: Optimization of Human-Robot Teams in Hospital Logistics Operations

Odense’s University Hospital (OUH) will benefit from a reactive AI-based resource management and scheduling system for material transport logistic operations. The main goal is to improve upon current task management systems with the inclusion of an AI-driven optimized scheduler that will be able to oversee all the available robots and to plan, schedule and assign tasks to the relevant hospital workforce, mainly logistic robots but also employees. The proposed task management software will have several functions and therefore will contain several different conceptual elements: (1) an automated task-generation system, based on Reinforcement Learning (RL) algorithm, that analyzes the relationship between room use and materials requirements to predict what will be needed where and when based on past experience; (2) a scheduling element that knows what transport resources are available to it, their status and where they are; and can create an optimal schedule out of transport requests generated from user input or the task generation above; (3) a reactive planning element that will rework the schedule regularly, e.g., either every hour or when new on-demand transport requests are received; (4) a transport optimizing element that analyzes the efficiency of the transport and adjusts scheduling parameters to produce maximal transport for minimal energy use and minimal task requests to humans; (5) a route generator element that creates efficient routes for the robots and sends these to robots with their new tasks, in accordance with the schedule, coupled with a route status analyzer which takes input from sensors on the robots and around the hospital to determine the location of any blockages; (6) A sensory data analyzer that can use incoming data from various infrastructure sources to inform the decision-making elements, e.g., use of elevator position to inform the route generator or use of smart cameras that can measure room occupancy for the task generator; (7) A representation of (a) task criticality, i.e., planned, urgent and critical in emergency situations, (b) the current status of the material flow, (c) the robots (name, capabilities, location, current task and status) and (d) item transport requests (also available in a form readable by humans); (8) and a supervision element that will be utilized to identify and criticize any suboptimal decisions made by the scheduler and provide feedback that will be used as input for a reinforcement learning sub-component. Task and material flow reports collected and shared by the hospital service and logistics departments of OUH, currently exceeding 555,000 entries describing various material flow logistic cases, i.e., transfer of medication, healthcare equipment and samples, will provide a variety of types of inputs and tasks. The system could automatically obtain information from various hospital software sources, e.g., human workforce positions provided by the proposed event-based messaging system by updating and adapting the current emergency messaging solution elevator status and sensors in the hospital.

### 3.11. Use Case 11: Co-Development and Evaluation

Bayındır Hospital Söğütözü in Ankara is one of the three high-capacity hospitals that belongs to Bayındır Healthcare Group. Bayındır Healthcare Group have three hospitals, one medical center and seven dental clinics. All healthcare facilities material management system can be centrally monitored and controlled. This provides an additional opportunity to study the impact of planned AI implementations over multi-location inventory systems. The hospital has specific experiences and requirements regarding healthcare logistics. It has an existing barcode scanning system for collecting healthcare and inventory information that aggregates centrally for the planning the availability of medical supplies and logistics management. However, the hospital may still benefit from a new picture recognition and AI-based system in terms of time savings, reductions in human error, and an increase the safety by reducing the contact between the healthcare staff and patients. Furthermore, material management and operation room scheduling are highly interrelated in practice. Using the OR schedules to trigger the purchase of perioperative materials is expected to further reduce inventory costs and increase operational efficiency compared to independent material management systems [39]. In a comparison to standalone applications of automated inventory tracking, predictive logistics, and cognitive automation, an additional understanding of the impact of integrated AI applications on healthcare logistics operations will bring several challenges, including data storage and management, data exchange, security and privacy, and integrated decision-making.

## 4. Discussion: Benefits and Challenges for AI in Hospitals

The specific benefits and data as well as AI application challenges are presented and discussed in this section, based on the outlined case studies and additionally directed towards the contribution against pandemic situations, such as COVID-19.

The use cases presented in Table 3 are distinguished by specific aspects often related to the area of interest, e.g., diagnosis, care, treatment, logistics or rehabilitation, or to the targeted goals, e.g., increase the efficiency of a certain health care process, improve its quality, or increase the service level. However, the detailed description of the aforementioned case studies suggests how all the involved hospitals are affected by common challenges and potential barriers to the adoption of AI to their healthcare processes on regular basis. In particular, it is possible to define three main issues which should be properly managed to ensure an efficient and effective adoption of AI tools and techniques in the healthcare delivery processes which distinguish European hospitals. The first aspect to be considered is the human acceptance and the real adoption of AI solutions in hospitals. The resistance to automated and partially obscure tools which offer assistance in several healthcare services is a major obstacle to overcome. Leveraging such tools in traditional diagnosis, care and treatment processes is useful but often distinguished by a low level of trust, in particular by doctors and medical personnel. Furthermore, the usage of such AI solutions should not increase the complexity or time required to complete certain medical process, therefore offering an adequate and well-designed interaction with human adopters. The second challenge to be tackled to foster the adoption of AI in European hospitals is the proper management of medical data. This information is distinguished by some features which make their storage and usage much more sensitive than other data typically collected in digital environments.

**Figure 2:**
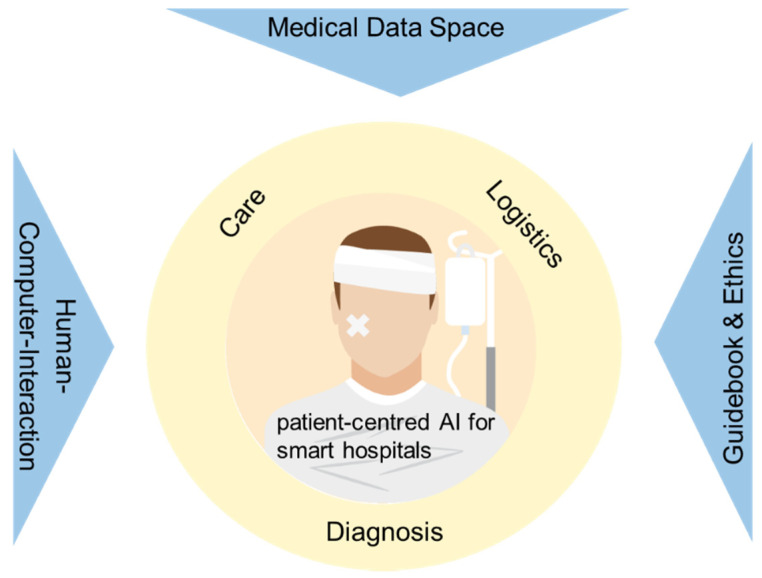
Linkage between transversal activities and application areas for AI adoption in European hospitals.

However, as COVID-19 dramatically revealed, the value beyond medical data is huge. In particular, the opportunity to systematically collect data concerning the patient conditions, made diagnosis, performed treatments and defined care offer to the hospitals of the future the chance to significantly increase the efficacy and efficiency of the healthcare services delivered. The last area involved by AI structural adoption in European hospitals deals with technology selection and ethics. The former includes the complex and interrelated process of selecting a novel technology for its adoption in healthcare services, as represented by the solutions based on AI algorithms. The assessment of the most appropriate AI based technology to be adopted to ease diagnosis, treatment or care activities is a complex and distinguished by uncertain and multiple feasible outcomes with different and contrasting scenarios. The latter deals with the ethical aspects involved in the adoption of AI tools and techniques, from machine based medical decision to personalized treatments, from sharing of personal health data to acceptance of robot medical personnel. Finally, a latter aspect concerning the challenges of adopting AI in hospitals necessarily has to be mentioned, e.g., the appropriate involvement of adequate stakeholders. Indeed, this last issue is of fundamental importance to ensure the real usage of AI-based solutions in daily hospital activities by doctors, acceptance of renovated treatments and procedures by patients as well as commitment by local administrators to this modern form of health care assistance. Therefore, the process of stakeholder commitment is of paramount importance and should be adequately planned and implemented. Considering all the abovementioned challenges and potential obstacles, the following paragraphs propose possible solutions to overcome these difficulties, to ensure the adoption of AI solutions in European hospitals and maximizing the efficacy of the innovation provided. In particular, the proposed actions are grouped into three categories, human–computer interaction, medical data space, and guidebook and ethics. The linkage between these transversal activities with the application areas proposed in the manuscript is presented in the following Figure 2.

*Human–Computer-Interaction*: Despite progress in the field of health care data analytics, resulting in more and more prototypes and technical advancement, actual adoption by key stakeholders such as doctors remains low [28,29]. This aspect will rise in relevance when the respective systems increase in intelligence and analytical capability. Accordingly, an increased focus on human–computer interaction spanning pre-design, design and post-design phases as well as catering to user, system, task and interaction characteristics [30] holds the potential to increase AI adoption and user satisfaction in clinical practice [31].

*Medical Data Space*: In addition, data connections in a Medical Data Space (MDS) with distributed AI applications will help to share resources and to support specially and severely affected regions and hospitals. In additions, overall data transparency and analysis will help to fight virus outbreaks earlier through faster detection and containment options due to AI analysis. The Medical Data Space (MDS) is a specialization of the International Data Space (IDS), which provides a trustworthy, secure and cross-domain data space allowing to build an economy of data between companies of all domains and sizes. IDS was the result of R&D activities in 2015 and is now actively promoted through the Industrial Data Space Association. It is in cooperation with the OPC foundation, the FIWARE foundation and the Industrial Value Chain Initiative and the Platform Industry 4.0. The IDS and thus the MDS define an architecture of data providers and consumers, which are linked through connectors forming the data space. The architecture is defined in the IDS document describing the layers of the architecture model which in turn describe the key components necessary to realize a data space [40]. The first prototype has been presented in 2018 at the Hannover fair. The MDS concept targets the connectivity of local data spaces in hospitals for analytics and the application of AI-based algorithms for research or hospital internal use. Therefore, special services are necessary to not only store and manage the transfer of medical data securely and maintaining the sovereignty of the data owner, but it must additionally conform to requirements on anonymity and protection of personal medical data sets. Here, the element of value-added services for the data space becomes relevant enabling pseudonymization and anonymization features in the process.

Medical data of patients is a highly sensitive and therefore regulated asset which requires handling in a secure and protected environment. The Medical Data Space (MDS) builds upon the international data space to deliver a secured, controlled data storage and processing environment to build an economy of data between providers and consumers retaining sovereignty and control. The MDS extends this to address the additional medical constraints. They key concept in MDS is the trusted connector which links both parties and enforces the security and privacy policies defined. In addition to access management the MDS architecture introduces data-processing services (data-apps) which can preprocess data before or after transfer. As AI-driven smart hospitals rely basically on data targets the connectivity of local data spaces in hospitals for analytics and the application of AI-based algorithms for research or hospital internal will be used. Therefore, special services are necessary to not only store and manage the transfer of medical data securely and maintaining the sovereignty of the data owner, but it must additionally conform to requirements on anonymity and protection of personal medical data sets. Here the element of value-added services (data-apps) for the data space becomes relevant enabling specifically pseudonymization and anonymization features in the process. In future works, we plan to demonstrate that medical data space technology can provide the foundation for the development and deployment of novel AI and data management data-apps. Specifically, a pilot program for the analysis and management of in-hospital cardiac patient intervention treatment with the goal of understanding and analyzing several key factors that impact the ability and capacity of a hospital to provide treatment. The location for this future installation will be the Evaggelismos Hospital in Athens.

*Guidebook and Ethics*: There is clear evidence of the relevance of organization and management of the technological issue in the health care, concept further reinforced on the light of recent COVID-19 pandemic [41]. Assessment, supply, prioritization, appropriate usage and exploitation are indeed not trivial duties, and the final success of any health process is widely affected by technology management issues. In the modern re-setting of health-care delivery via technology innovation, data driven management, health technology assessment, clinical practice guidelines as well as medical leadership are the main topics that have to be addressed [42]. Knowledge management and technology innovation with their continuously growing potentiality can indeed transversally represent the answer to the demand of efficacy and efficiency of the system. Furthermore, great expectations are placed in information and communication technologies (ICT) with their contribution in the development of eHealth and closely in AI with its paramount applications in the various sectors of medical practice and public health. The change in clinical practice through and by means of the injection of technological innovation is today decisive to make the health and care systems able to face to the continuous economic, socio-demographic and epidemiological pressures [17]. However, technological innovation, although important and central, must be carefully examined and accompanied to ensure that it really corresponds to effective social innovation [43]. Furthermore, as really recently underlined by a joint report of EIT Health and McKinsey [44]. AI has indeed many potentialities for the improvement in care outcomes, patient experience and access to healthcare services. AI is thought to increase productivity and the efficiency of care delivery and allow healthcare systems to provide more and better care to more people. Finally, it can support the faster delivery of care, mainly by accelerating diagnosis time, and help healthcare systems manage population health more proactively, dynamically allocating resources to where they can have the largest impact and need. As addressed by MedTech Europe, developing AI systems and algorithms for healthcare settings requires specific skillsets which are in short supply, and investment in education and training of professionals involved (e.g., data scientists, practitioners, software engineers, clinical engineers), is mandatory [18].

Ethical issues are a major hurdle to full-scale AI application use as many cases might bring about risks such as wrong diagnosis or deviant therapy, as well as dissent among personnel due to different opinions regarding correct AI analysis and advice. Therefore, not only HCI issues but also human-human interaction and collaboration issues and ethical questions to be solved and communicated among people first of all before AI can contribute according to the full potential in health care.

## 5. Outlook

AI will play a significant role in future hospital health care systems. Applications such as ML will further advance the development of processes in several fields inside the hospital, of which we focus in medical diagnosis, logistics and care in this article. Important obstacles remain, such as regulations, integrations to the Electronic Health Record (EHR), standardization, medical devices certificates, training professionals, costs, updates—but this is manageable. It is important to stress that AI applications will not replace human clinicians but help them to concentrate on important human-related processes and to make correct diagnoses with less analysis and decision time. This hopefully provides them with time and focus to support patients from a specific human perspective. As a result of the developments in computational power and algorithmic advancements, combined with digitalization and improvements in data collection methods and storage technologies, the healthcare sector today is supported by AI, ML and robotics as never before in the history of medicine. Besides monitoring large-scale medical trends, these new technologies also allow measurement of individual risks based on predictions from big data analysis. AI has a key function in the healthcare management of the future. Research has already proven the game changing potential of AI in various fields of healthcare, such as those outlined in the use cases in this article. AI-based methods have been successfully developed to address several healthcare logistics problems such as appointment planning, patient and resources scheduling, resource utilization, and predicting demand for emergency departments, intensive care units, or ambulances [45]. In addition, there already exist a number of research studies which suggest that AI can perform at least as good as humans at basic healthcare functions, such as diagnosis. Today, malignant tumors are spotted more successfully by algorithms than humans [46]. As a consequence of rapid technological advancements, combined with ML’s enhanced ability to transform data into insight, many of the medical tasks previously limited to humans are expected to be taken on by algorithms [47]. However, there are several reasons why it will take a long time before AI might take over comprehensive fields of activity from humans in hospitals and healthcare: recent developments show that AI systems will not replace humans on a large scale, but rather will support them in their efforts of patient care. Progressing into future times, healthcare specialists can switch to tasks and job designs focusing on unique human skills such as empathy and care. One risk within this development might be the position of healthcare providers who are unable or refuse to work in collaboration with AI applications, endangering their contributions and jobs. The most important obstacle regarding AI applications in healthcare are not the capabilities or benefits of the technologies themselves, but their applicability in medical practice. Widespread use of AI systems requires approval by regulating institutions, integration with existing systems, sufficient standardization with similar products, training of healthcare professionals, and solutions regarding issues of data privacy and security. These challenges will eventually be solved, but it will take significant time and resources [46]. The COVID-19 crisis has revealed the challenges for healthcare systems—also for future pandemic situations. This increased attention to the potential of AI in healthcare as one means of pandemic management and prevention. Major challenges in responding to COVID-19, such as managing limited healthcare resources, developing personalized treatment plans, or predicting virus spread rates, can be addressed by recent developments in AI and ML. Wynants et al. [48] have already listed 31 prediction models in a review of early studies of COVID-19. The prospective post-COVID-19 era in preparation for future pandemic events will likely feature advanced healthcare solutions in combination with operation research modeling [49]—and AI will be a crucial part of it as outlined in this paper with 11 use case studies from European hospitals. The challenges connected to such AI applications such as data management (HCI) have to be addressed soon in order to prepare hospitals for future challenges, e.g., pandemic situations [50]. This is a core challenge for health care management science and the implication for hospital practice in order to apply the full potential of AI and ML to health care systems [51].

## Figures and Tables

**Table 1:** Included survey and case study hospitals in Europe.

Hospital	Type/Inpatient per Year/Outpatient per Year/Beds ^1^	Main Health Area(s)	Specific Application Areas (AA)/Focus on (F)/Expected Output (EO)
University Hospital of Bern (Switzerland)	Public/6000/737,830/64 (2018) ^2^	The University Clinic for Obstetrics and Gynecology of Inselspital	AA: Diagnosis F: Fetal state assessment during labor EO: AI-based decision support system for fetal state assessment during labor. The solution can assist obstetricians in accurately assessing the fetal state in clinical practice during labor.
Kuopio University Hospital (Finland)	Public/99,000/517,000/590 (2019) ^3^	All branches	AA: Diagnosis F: Finding new diagnostic and treatment methods, for coronary artery disease. EO: An AI-based decision support system for selecting those patients among suspected CAD who benefit from further imaging.
Hospital of Bozen (Italy)	Public/25,064/737,830/697 (2018) ^4^	All branches	AA: Care F: Rheumatological diseases and diabetes EO: An intelligent tool able to support the definition and scheduling of the different laboratory tests, medical examinations and hospitalization.
La Fe University Hospital (Spain)	Public/45,062/148,702/1004 (2019) ^5^	Management of Chronicity (Integrated Care) and Active and Healthy Aging	AA: Care F: Strategic initiatives on integrated care for patients with complex chronic and/or oncological conditions EO: An intelligent tool able to improve the management of chronic patients and to characterize the use of resources throughout chronic patients’ healthcare, reducing the economic burden for hospitals.
Federico II University of Naples (Italy)	Public/n.a./365,000/1000 (2019) ^6^	Arterial hypertension on the cardiovascular system	AA: Care F: Arterial hypertension with particular reference to ischemia heart disease EO: Development of diagnostic and therapeutic methodologies in the field of cardiac rehabilitation; development of remote monitoring systems (telemedicine) for patients with high cardiovascular risk.
Orton Ltd., The Private Unit Helsinki Univ.Hospital (Finland)	Private/2000/22,000/40 ^7^	Orthopedics, neurosurgery, cancer treatment, pain medicine and rehabilitation	AA: Care F: Ethical, rehabilitation and preventive care EO: Developing new tools for the treatment and rehabilitation of musculoskeletal disorders and other conditions.
Odense University Hospital (Denmark)	Public/104,229/1,104,229/1038 (2019) ^8^	All branches	AA: Logistics F: Future of health care in mind, incorporating innovative clinical and logistical technologies. EO: To serve as a test bed for new medical technology, including an extensive use of robotics and AI.
Bayındır Hospital (Turkey)	Private/11,284/252,995/131 (2019) ^9^	All branches	AA: Logistics F: Materials management and scheduling EO: Optimizing resource allocation and medical materials planning, reducing operational costs and patient waiting times
University Hospital Essen (Germany)	Public/50,000/195,000/1300 (2019) ^10^	Genetic medicine, immunology, oncology, cardiovascular medicine and transplants	AA: Logistics//F: Care operations with materials management and supply//EO: Digitalized, patient- and employee-oriented organization. To minimize time spent for the nurses on documentation and administrative tasks to allow more time for direct patient care.

^1^ Data from hospital sources. Definitions might differ due to national data regulations. ^2^ University Hospital of Bern: http://www.frauenheilkunde.insel.ch/de/ueber-die-klinik, accessed on 2 October 2020. ^3^ Kuopio University Hospital: https://www.psshp.fi/web/en/organisation/operations-and-tasks, accessed on 2 October 2020. ^4^ Südtiroler Sanitätsbetrieb: https://www.sabes.it/de/578.asp, accessed on 2 October 2020. ^5^ La Fe University Hospital: Hospital activity report, 2019. ^6^ Federico II University of Naples. ^7^ Orton Ltd. University Hospital. ^8^ Odense University Hospital: https://en.ouh.dk/about-ouh/key-figures, accessed on 2 October 2020. ^9^ Bayındır Hospital. ^10^ Universitätsklinikum Essen: https://www.uk-essen.de, accessed on 2 October 2020.

**Table 2:** Included survey and case study hospitals in Europe.

Heath Organization	Current Problems and Approach	Vision on Potential Application of AI	Expected Improvement—KPIs
University Hospital of Bern, Department of Obstetrics and Gynecology	Fetal assessment based on Cardiotocography (CTG) or electronic fetal monitoring (EFM) limitations.	Their vision is to develop a medical decision support system, which can assist obstetricians in accurately assessing the fetal state in clinical practice during labor.	Improvement of decision-making can improve fetal outcomes after delivery and avoid unnecessary medical interventions and their health implications for mother and fetus, as well as their economic implications. The KPIs of the AI application are: Fetal outcomes, measured by clinical adaptation (APGAR score) and hypoxia (measured by arterial pH), Invasive interventions for prematurely ending the delivery process, such as instrumental delivery or cesarean section, Economical costs of delivery.
Kuopio University Hospital	Currently, the diagnosis of coronary heart disease has changed towards the non-invasive imaging, which has led to increasing number of patients scheduled to CCTA. Interpretation of CCTA is affected by the image quality, experience of the doctor and by other issues, which can in terms lead to unnecessary repeated or additive diagnostic imaging.	The motivation is to develop an automatic AI-based analysis system for the coronary computed tomography angiography (CCTA): To enhance diagnostic accuracy of CCTA and to guide clinical decision making. Interpretation of CCTA will be systematically guided by the standard AI-based analysis system.	The patients need only one diagnostic method and the workflow of the interpretation of CCTA become more fluent. Relevant KPIs are: Increased number of CCTA imaging in one center, Improved patient convenience, safety and decreased health care costs, Improved effectiveness leads to shorter waiting times and shortened queues.
Hospital of Bozen	Limitations on healthcare resources management and chronic care pathways definition	AI tools to support the definition and scheduling of the different laboratory tests, medical examinations and hospitalization which affect STHA patients, personnel, equipment and resources inside and outside the hospital and located in multiple areas of the geographical territory of its responsibility	Ease the management of healthcare resources with a particular focus on rheumatological diseases and diabetes as chronic diseases. Relevant KPIs are: Decrease waiting time to access to scheduled medical examinations and labor tests, Average cost to provide the healthcare services to the chronic care population, Quality of the medical treatment, e.g., percentage of re-hospitalized patients.
La Fe University Hospital	Chronic diseases (CDs) represent the major cost of morbidity and mortality and lead to 86% of all deaths. In Europe, these account for more than 75% of the healthcare burden with a cost for the economy of €700 billion per year.	AI will help to: Improve the management of chronic conditions and multimorbidity in the face of aging population and its implication on public health; Contain the impact and global burden of chronic conditions, multimorbidity and frailty on individual quality of life and on healthcare systems; Strength the clinical management of complex chronic conditions and multimorbidity having a better understanding of the individual prognosis and disease evolution, and targeting personalized interventions.	Optimization of resources and the clinical flow of chronic patients at Hospital. Relevant KPIs are: Efficiency on the allocation and consumption of resources, Right assignment of chronic patient to care pathway, Decrease in turnaround time, Selection of right pathway, Avoidable episodes of care inadequate use.
Federico II University of Naples	Today CVD is the leading cause of death in Europe; presently 47% of all deaths in Europe and 40% of all deaths in the European Union (EU) are attributable to CVD. This means that across Europe as a whole 4 million deaths per year currently occur due to CVD, of which 1.9 million are in the European Union	Use of AI may help clinicians in problem solving and patient’s management. AI process may be used to improve process of health care management with specific regards to resource allocation, patient management.	Rapid assessment of correct management strategy. Relevant KPIs are: Improvement of timeliness in critical event treatment, Reduction of ambulatorial visits, Forecasting of avoidable critical conditions.
Odense University Hospital	Maintain high quality treatment for our patients in a demographic development scenario and increasing chronic conditions	Need to rely on AI and robots to ensure quality level and improve security in repetitive tasks, while alleviating staffing challenges.	Optimize handling of transports and logistics. Relevant KIPs are: Improve timing for transportation of patients or samples. Release of staffing resources to other tasks/areas. As well as an improved working environment for staff.

**Table 3:** AI Use Cases, AI Methods and Outcomes.

Use Case	Objectives	AI Method	Data Available	Defined Outcomes	Contributions against Pandemic Situations
Diagnosis (1) MDS for Coronary Artery Disease (CAD) diagnosis	The aim of this study is to train a ML algorithm to distinguish patients with suspected CAD to those who benefit from further imaging studies and to those who don’t. In other words, to evaluate the risk of the patient to have prognostic CAD for customized clinical decision-making.	Disease State Index (DSI), which is a method to quantify the probability to belonging to a certain disease population, originally developed to support clinicians in diagnosing Alzheimer’s Disease [34]. It is designed to be ‘disease-agnostic’, so that it can be used equally well for other diseases, provided that data are available.	For the study, a number of contemporary CCTA studies imaged in Kuopio University Hospital (KUH) as well as ECG, myocardial perfusion, invasive coronary angiography imaging and essential clinical data (age, sex and other demographic data, medical history, cardiovascular risk factors and medication) are gathered from existing clinical databases in KUH.	Algorithms and AI solutions for doctors supporting clinical decision making in CAD diagnosis.	Reduction of visits to the hospital, which increases the patient and personnel safety.
Diagnosis (2) AI based automatic arrhythmia analysis	In this use case, state-of-the-art artificial intelligence (AI) based arrhythmia analysis algorithms are developed and integrated into wearable sensors. Development of novel AI-based arrhythmia monitoring system aims to improve arrhythmia detection: Enable longer non-invasive monitoring time.	State-of-the-art AI based arrhythmia analysis algorithms are developed and utilized to atrial fibrillation (AF) screening in patients with transient ischemic attack (TIA) or cryptogenic stroke (CS) and detection of post-operative atrial fibrillation in cardiac surgery patients. Used methods: neural networks, deep learning, ML.	6000 24 h Holter recordings with arrhythmia annotations. Wearable sensor database: 700 patients (300 patients with AF episodes) with wearable sensors. New: TIA/CS database is collected: 48h home monitoring of simultaneous wearable PPG and ECG-recordings from 100 TIA/CS patients.	Developed AF-screening solution will enable long arrythmia monitoring time and increased rate of AF diagnosis. Wearable sensors offer improved patient usability and AI assisted arrythmia diagnosis requires minimal effort from healthcare professionals; AF diagnosis has important impact to patient itself, since anticoagulation may save the patient’s life (prevent cardioembolic stroke). Cost saving potential: one prevented stroke can save 120,000€ to society.	Reduction of visits to the hospital, which increases the patient safety. Possibility to assess arrythmia of corona patients remotely. Increases patient and personnel safety.
Diagnosis (3) Medical decision support system for fetal assessment during labor	Improving fetal assessment with accurate prediction of fetal hypoxia and reduction of caesarean and instrumental delivery rates. Develop an AI-powered clinical decision support system.	Ensemble methods (e.g., stacking and blending) combining Explainable AI (aka XAI), neural networks (e.g., CNN and RNN), and gradient boosting techniques (e.g., XGBoost)	The maternity ward of the Department for Obstetrics and Gynecology in the University Hospital of Bern will provide a dataset of cardiotocographic (CTG) recordings. It includes physiological data such as maternal heart rate, fetal heart rate, contraction strength. The dataset is labelled by MDs.	The AI will focus on removing the existing great inter- and intra-observer variability while achieving at least the same average accuracy as medical doctors following the “Updated 2015 FIGO Intrapartum Fetal Monitoring Guidelines”. The integration of our AI-powered system should lead to a direct and positive impact on effectiveness and efficiency.	Assisting personnel in diagnosis with AI in a situation where there are not enough experienced personnel available due to the pandemic.
Diagnosis (4) Diagnosis in Epidermolysis bullosa, a rare genetic disease	To support disease prediction and diagnosis through the integration of extensive biological data (images, genomics, molecular) and epidemiological (immunological, clinical, demographic, lifestyles) to identify genomic lesions, proteins and immune-logical states associated (biomarkers).	ML algorithms will be trained to integrate phenotypic and clinical data to improve accurate prediction of progress of Epidermolysis bullosa. AI-based methods will also be used for disease comprehension and therapeutic target selection by unravelling the affected genetic and molecular players and pathways.	This use case will exploit data, competencies, and facilities of the Modena EB-Hub, the center for diagnosis, research, assistance and development of innovative therapies created in January 2020 at the General Hospital of Modena.	Definition of AI-based decision support systems to expedite diagnosis, correct misdiagnosis, diagnose previously undiagnosed, and stratify EB patients for advance therapeutic intervention through the integrative analysis of clinical phenotypes and patient health records, genetic information, molecular levels, biochemical fingerprints and patient images.	Assisting doctors’ in the diagnostic process during the pandemic, when the resources to be used for diagnosis is limited. Maintaining normal procedures of diagnosing other health problems during the pandemic.
Care (5) Chronic care pathway and resources characterization, simulation of demand and prognosis.	AI techniques applied to analyze the pathways of chronic care patients providing simulation and prediction capacities about the demand of use of hospital services and resources	ML techniques (neuronal networks; LSTM; statistics predictions modeling; random forest; decision trees). AI adjustment to chronic care attention, prototype testing, application evaluation (KPI).	Historical clinical records for patients with chronic diseases. Data about care plans and use of hospital services and resources (pathways) made by this group of patients based on degree of frailty. Macro parameters from population (estimate demand/prognosis)	AI agent and tool for dimensioning demand of resources, including prognosis and simulation, both at individual and population level. Intelligent assistant for redefinition/optimization of care plans	Reduction of the transmission risks by being able to re-organize the pathways according to pandemic context.
Care (6) Critical Conditions identification and prevention	Identification and prevention of critical conditions: Analysis of vital signs, automatic recognition of symptoms (e.g., skin rash, mood change) and direct interaction with patients.	Machine Learning Techniques such as DNN, Reinforcement Learning, Natural Language Processing and Statistical Methods.Adjustment chronic care, prototype, evaluation (KPI).	Test of algorithms in hospital of Bozen with either live settings or retrospective data. Retrospective data as heart rate, respiration rate, oxygen saturation and blood pressure. Moreover, general data such as age, sex, weight, height and other diseases.	AI tool for critical conditions identification and prevention along the chronic care pathway	Control of patients with COVID-19 confined to their homes, before variations in their critical conditions. Increase in patient and family safety, especially in patients with COVID-19 who live alone.
Care (7) Intelligent resources management	An intelligent algorithm is developed to efficiently manage the scheduling of hospital resources.	Evolutive, self-learning and auto-adaptive techniques focused on chronic care, prototype testing, validation through KPI.	Hospital models of processes for resource utilization. Information: processes, cost, service level, delivery time, resource utilization, medical personnel qualification.	Scheduling planning tool for optimal management of hospital care resources for patients with chronic diseases.	Reduction the transmission risks. Better planning of resources in compatibility with pandemic demand.
Care (8) Monitoring of the recovery process	Remote determination of vital parameters such as heart rate and respiration rate for an improved recovery monitoring.	Methods in the Area of computer vision and ML i.e., CNN, BNN, adaptive optical flow, SVM etc.	Recordings from lab situations available; more data will be generated within the Fraunhofer InHaus-Centre, Test of algorithms in hospital of Bozen	Software for vital parameters. Transfer to hospital environment; continuous monitoring; fast obstacle identification; safe solution; contactless	Reduction the transmission risks in professionals by reducing contact with monitored admitted patients with COVID-19.
Logistics (9) Material consumption recognition and prognosis	Develop an automatic material documentation on the care wagon or the material store in the nursing ward based on computer vision. A material consumption prognoses is developed with the derived data.	ML (computer vision, CNN): Used materials are matched with patient cases and their diagnoses and treatments. Thus, it is known which and how many materials are needed by the individual patient cases.	- Material lists - Master and movement data of the materials (order history) - Demographic patient data (gender, age, weight, etc.) - Patient treatment history - - Automatic stock updates for all materials on wagon	- Automatic material documentation and transport - Transparent material consumption for individual patient cases - Specified case cost calculation - Higher planning reliability for material orders - - Immediate reaction to material shortage	- Improved forecasting for pandemic related uncertanities - Dynamic management of limited material (such as masks, protective visors and clothing, antiseptics, etc.) by predicting patients’ disease trajectory
Logistics (10) Optimizing logistic operations	Optimize the internal logistics operations of the hospital by considering both manual and automatic transport in a resource management and scheduling framework. Generate recommendations for how to improve manual and robotic logistics, based on gathered data.	Reinforcement learning (multi-agent motion and path planning)	- Hospital maps - Data (sensor data, operational data) from robots operating at the hospital - Data from the hospitals material management system - Generating data from current hospital sensor infrastructure - Knowledge about areas that are frequented by visitors or patients probably infected with COVID-19	- Status reports for certain characteristics of automated and manual logistics operations - Recommendations for optimization of material transport - Better understanding of the events leading up to an incident report (e.g., materials arrived late, or robot stopped unexpectedly) - Facilitate future integration of robotic solutions in hospitals - Automatic avoidance of infections areas (e.g., areas frequented by visitors)	- Decreasing transmission risks to healthcare providers by minimizing the patient contact. - Optimal management of critical resources such as Intensive Care Unit (ICU) beds.
Logistics (11) Co-development and evaluation	Integration of optimization of internal logistics operations and material consumption	Predictive analytics and cognitive automation	- Material lists - Master and movement data of the materials - Demographic patient data - Patient treatment database - Availability of healthcare resources	- Adaption routines and experiences, e.g., comparison of material recognition with barcode system (already existing, comparative case) - Management of resources in multi-location setting	- Centralized planning of material consumption and shortage - Optimal assignment/scheduling of critical resources (healthcare personnel, ICU, operation rooms, etc.)

## Data Availability

Not applicable.

## References

[1] Halawa F., Madathil S.C., Gittler A., Khasawneh M.T. (2020). Advancing evidence-based healthcare facility design: A systematic literature review. Heal. Care Manag. Sci..

[2] McKee M., Merkus S., Edwards N., Nolte E. (2020). The Changing Role of the Hospital in European Health Systems.

[3] Increase in the Share of the Population Aged 65 Years or Over Between 2009 and 2019. https://ec.europa.eu/eurostat/statistics-explained/index.php?title=Population_structure_and_ageing.

[4] Michal J., Ecarnot F. (2020). The shortage of skilled workers in Europe: Its impact on geriatric medicine. Eur. Geriatr. Med..

[5] Moser E., Narayan G. (2020). Improving breast cancer care coordination and symptom management by using AI driven predictive toolkits. Breast.

[6] Abràmoff M.D., Tobey D., Char D.S. (2020). Lessons Learned About Autonomous AI: Finding a Safe, Efficacious, and Ethical Path Through the Development Process. Am. J. Ophthalmol..

[7] Wood D.A., Mahmud E., Thourani V.H., Sathananthan J., Virani A., Poppas A., Harrington R.A., Dearani J.A., Swaminathan M., Russo A.M. (2020). Safe Reintroduction of Cardiovascular Services During the COVID-19 Pandemic. J. Am. Coll. Cardiol..

[8] McKinney S.M., Sieniek M., Godbole V., Godwin J., Antropova N., Ashrafian H., Back T., Chesus M., Corrado G.S., Darzi A. (2020). International evaluation of an AI system for breast cancer screening. Nat. Cell Biol..

[9] Arbabshirani M.R., Plis S., Sui J., Calhoun V.D. (2017). Single subject prediction of brain disorders in neuroimaging: Promises and pitfalls. NeuroImage.

[10] Bzdok D., Meyer-Lindenberg A. (2018). Machine Learning for Precision Psychiatry: Opportunities and Challenges. Biol. Psychiatry: Cogn. Neurosci. Neuroimaging.

[11] Lee E.-J., Kim Y.-H., Kim N., Kang D.-W. (2017). Deep into the Brain: Artificial Intelligence in Stroke Imaging. J. Stroke.

[12] Awan S.E., Sohel F., Sanfilippo F.M., Bennamoun M., Dwivedi G. (2018). Machine learning in heart failure: Ready for prime time. Curr. Opin. Cardiol..

[13] Hampe N., Wolterink J.M., Van Velzen S.G.M., Leiner T., Išgum I. (2019). Machine Learning for Assessment of Coronary Artery Disease in Cardiac CT: A Survey. Front. Cardiovasc. Med..

[14] Esteva A., Kuprel B., Novoa R.A., Ko J., Swetter S.M., Blau H.M., Thrun S. (2017). Dermatologist-level classification of skin cancer with deep neural networks. Nature.

[15] Gulshan V., Peng L., Coram M., Stumpe M.C., Wu D., Narayanaswamy A., Venugopalan S., Widner K., Madams T., Cuadros J. (2016). Development and Validation of a Deep Learning Algorithm for Detection of Diabetic Retinopathy in Retinal Fundus Photographs. JAMA.

[16] Mishima H., Suzuki H., Doi M., Miyazaki M., Watanabe S., Matsumoto T., Morifuji K., Moriuchi H., Yoshiura K.-I., Kondoh T. (2019). Evaluation of Face2Gene using facial images of patients with congenital dysmorphic syndromes re-cruited in Japan. J. Hum. Genet..

[17] Global Strategy on Human Resources for Health: Workforce 2030. https://apps.who.int/iris/bitstream/handle/10665/250368/9789241511131-eng.pdf?sequence=1.

[18] Artificial Intelligence in Medical Technology: Delivering on the Promise of Better Healthcare in Europe. https://www.medtecheurope.org/wp-content/uploads/2019/11/MTE_Nov19_AI-in-MedTech-Delivering-on-the-Promise-of-Better-Healthcare-in-Europe.pdf.

[19] Xu S., Hu C., Min D. Preparing for the AI Era Under the Digital Health Framework. Proceedings of the 2019 ITU Kaleidoscope: ICT for Health: Networks, Standards and Innovation (ITU K).

[20] Tortorella G.L., Fogliatto F.S., Mac Cawley Vergara A., Vassolo R., Sawhney R. (2019). Healthcare 4.0: Trends, challenges and research directions. Prod. Plan. Control..

[21] Haluza D., Jungwirth D. (2015). ICT and the future of health care: Aspects of health promotion. Int. J. Med. Inform..

[22] Zijm H., Klumpp M., Freitag M., Kotzab H., Pannek J. (2017). Future Logistics: What to Expect, How to Adapt. Dynamics in Logistics. Lecture Notes in Logistics.

[23] Klumpp M. (2018). Automation and artificial intelligence in business logistics systems: Human reactions and collaboration re-quirements. Int. J. Logist. Res. Appl..

[24] Giusti R., Manerba D., Bruno G., Tadei R. (2019). Synchromodal logistics: An overview of critical success factors, enabling tech-nologies, and open research issues. Transp. Res. Part E Logist. Transp. Rev..

[25] Cardoen B., Demeulemeester E., Beliën J. (2010). Operating room planning and scheduling: A literature review. Eur. J. Oper. Res..

[26] Tuwatananurak J.P., Zadeh S., Xu X., Vacanti J.A., Fulton W.R., Ehrenfeld J.M., Urman R.D. (2019). Machine Learning Can Improve Estimation of Surgical Case Duration: A Pilot Study. J. Med Syst..

[27] Li F., Gupta D., Potthoff S. (2015). Improving operating room schedules. Heal. Care Manag. Sci..

[28] Kohli R., Tan S.S.-L. (2016). National University of Singapore Electronic Health Records: How Can IS Researchers Contribute to Transforming Healthcare?. MIS Q..

[29] Romanow D., Cho S. (2012). Straub Editor’s Comments: Riding the Wave: Past Trends and Future Directions for Health IT Research. MIS Q..

[30] Zhang P., Li N. (2004). An assessment of human–computer interaction research in management information systems: Topics and methods. Comput. Hum. Behav..

[31] Rzepka C., Berger B. User Interaction with AI-enabled Systems: A Systematic Review of IS Research. Proceedings of the International Conference on Information Systems.

[32] Preece J., Rogers Y., Sharp H., Benyon D., Holland S., Carey T. (1994). Human-Computer Interaction.

[33] Dix A., Finlay J., Abowd G., Beale R. (2003). Human-Computer Interaction.

[34] Mattila J., Koikkalainen J., Virkki A., Simonsen A.H., Van Gils M., Waldemar G., Soininen H., Lötjönen J., Initiative A.F.T.A.D.N. (2011). A Disease State Fingerprint for Evaluation of Alzheimer’s Disease. J. Alzheimer’s Dis..

[35] Ayres-de-Campos D., Spong C.Y., Chandraharan E. (2006). FIGO consensus guidelines on intrapartum fetal monitoring: Cardi-otocography. Int. J. Gynecol. Obstet..

[36] (2006). American Academy of Pediatrics, Committee on Fetus and Newborn; American College of Obstetricians and Gynecologists, Committee on Obstetric Practice. The Apgar Score. Pediatrics.

[37] Brasil S., Pascoal C., Francisco R., Ferreira V.D.R., Videira P.A., Valadão A.G. (2019). Artificial Intelligence (AI) in Rare Diseases: Is the Future Brighter?. Genes.

[38] Danial C., Adeduntan R., Gorell E., Lucky A.W., Paller A., Bruckner A., Pope E., Morel K.D., Levy M.L., Li S. (2014). Prevalence and Characterization of Pruritus in Epidermolysis Bullosa. Pediatr. Dermatol..

[39] Epstein R.H., Dexter F. (2000). Economic analysis of linking operating room scheduling and hospital material management in-formation systems for just-in-time inventory control. Anesth. Analg..

[40] Reference Architecture Model IOP Publishing Internationa Data Spaces. https://www.internationaldataspaces.org/wp-content/uploads/2019/03/IDS-Reference-Architecture-Model-3.0.pdf.

[41] Country & Technical Guidance—Coronavirus Disease IOP Publishing WHO. https://www.who.int/emergencies/diseases/novel-coronavirus-2019/technical-guidance.

[42] OECD (2019). World Health Organization Improving Healthcare Quality in Europe.

[43] Employment, Social Affairs & Inclusion IOP Publishing Europa. https://ec.europa.eu/social/main.jsp?catId=1022&langId=en.

[44] EIT Health, McKinsey & Company (2020) Transforming Healthcare with AI: The Impact on the Workforce and Organisations. IOP Publishing EIT Health. https://eithealth.eu/wp-content/uploads/2020/03/EIT-Health-and-McKinsey_Transforming-Healthcare-with-AI.pdf.

[45] Reuter-Oppermann M., Kühl N., Masmoudi M., Jarboui B., Siarry P. (2021). Artificial Intelligence for Healthcare Logistics: An Overview and Research Agenda, In Ar-tificial Intelligence and Data Mining in Healthcare.

[46] Davenport T., Kalakota R. (2019). The potential for artificial intelligence in healthcare. Futur. Heal. J..

[47] Noorbakhsh-Sabet N., Zand R., Zhang Y., Abedi V. (2019). Artificial Intelligence Transforms the Future of Health Care. Am. J. Med..

[48] Wynants L., van Calster B., Bonten M.M., Cllins G.S., Riley R.D., Heinze G., Schuit E., Dahly D.L., Damen J.A.A., Debray T.P.A. (2020). Prediction models for diagnosis and prognosis of COVID-19 infection: Systematic review and critical appraisal. BMJ.

[49] Niessner H., Rauner M.S., Gutjahr W.J. (2018). A dynamic simulation–optimization approach for managing mass casualty incidents. Oper. Res. Heal. Care.

[50] Klumpp M., Zijm H. (2019). Logistics Innovation and Social Sustainability: How to Prevent an Artificial Divide in Human–Computer Interaction. J. Bus. Logist..

[51] Bertsimas D., Orfanoudaki A., Weiner R.B. (2020). Personalized treatment for coronary artery disease patients: A machine learning approach. Heal. Care Manag. Sci..

